# Therapeutic role of adipose tissue–derived stem cells versus microvesicles in a rat model of cerebellar injury

**DOI:** 10.1111/jcmm.17083

**Published:** 2021-12-07

**Authors:** Nehad F. Mazen, Eman A. Abdel‐Fattah, Shimaa R. Desoky, Amal S. El‐Shal

**Affiliations:** ^1^ Medical Histology and Cell Biology Department Faculty of Medicine Zagazig University Zagazig Egypt; ^2^ Histology and Cell Biology Department Faculty of Medicine Suez University Ismailia Egypt; ^3^ Medical Biochemistry & Molecular Biology Department Faculty of Human Medicine Zagazig University Zagazig Egypt

**Keywords:** adipose stem cells, cerebellum, microvesicles, monosodium glutamate

## Abstract

Monosodium glutamate (MSG) is a controversial food additive reported to cause negative effects on public health. Adipose stem cells (ASCs) and their derived vesicles (MVs) represent a promising cure for human diseases. This work was planned to compare the therapeutic effects of adipose stem cells and microvesicles in MSG‐induced cerebellar damage. Forty adult healthy male Wister rats were equally divided into four groups: Group I (control group), group II (MSG‐treated), group III (MSG/ASCs‐treated), and group IV (MSG/MVs‐treated). Motor behaviour of rats was assessed. Characterization of ASCs and MVs was done by flow cytometry. The cerebellum was processed for light and electron microscopic studies, and immunohistochemical localization of PCNA and GFAP. Morphometry was done for the number of Purkinje cells in H&E‐stained sections, area per cent of GFAP immune reactivity and number of positive PCNA cells. Our results showed MSG‐induced deterioration in the motor part. Moreover, MSG increases oxidant and apoptotic with decreases of antioxidant biomarkers. Structural changes in the cerebellar cortex as degeneration of nerve cells and gliosis were detected. There were also a decrease in the number of Purkinje cells, an increase in the area per cent of GFAP immune reactivity and a decrease in the number of positive PCNA cells, as compared to the control. Rats treated with ASCs showed marked functional and structural improvement in comparison with MV‐treated rats. Thus, both ASCs and MVs had therapeutic potential for MSG‐induced cerebellar damage with better results in case of ASCs.

## 1 INTRODUCTION

Monosodium glutamate (MSG) is a salt originally derived from herb and commonly marketed as a flavour enhancer.^1^ It becomes extensively used in canned food, frozen entrees, crackers, processed meats and dietary supplements. It is also found in soaps, cosmetics, infant formula and vaccines.^2^

MSG dissociates in solution to glutamate and sodium ions.^1^ Glutamate is the foremost excitatory neurotransmitter in the body. Numerous transporters and receptors for glutamate are found in the gastrointestinal tract and nervous system. The intestine is the primary site for catabolism.^3^

The average daily intake of MSG is estimated to be 0.3–1.0 g in industrialized countries, which can be higher depending on the MSG content of food items and the individual taste preferences.^4^ Under normal conditions, acute toxicity of glutamate is uncommon. The oral dose that is lethal to 50% of subjects (LD50) in rats and mice is 15,000–18,000 mg/kg body weight.^5^

However, MSG has been tied to obesity and disorders in the respiratory, circulatory, reproductive and nervous systems.^6^, ^7^ It triggered symptoms, referred to collectively as ‘Chinese restaurant syndrome’ consisting of numbness at the back of the neck and arms, weakness and palpitations.^8^

The young and the elderly are most at risk from MSG as the blood‐brain barrier is not fully developed in the young and can be damaged by ageing or disease in the elderly. Ingredients that contain MSG are used in baby formula, allowing neurotoxins to be more accessible to the brain which is not yet well developed.^9^ Exposure of rats to MSG at the neonatal stage was found to cause severe damage of the hypothalamic nuclei resulting in increased body weight, fat deposition and decreased motor activity.^10^

Cell therapies are group of strategies that use live cells for healing aims. Their aims are to repair, replace and restore the biological functions of damaged tissue or organs. It can be applied in many clinical tests for the treatment of conditions as diabetes mellitus, liver disease and corneal, articular, neural and cutaneous lesions.^11^

Regenerative medicine is the process of regenerating or replacing the damaged cells, tissues and/or organs restore normal function. Tissue damage caused by degenerative diseases or neoplasia remains a vast challenge to treat.^12^, ^13^

Stem cells can differentiate into many different mature cell types such as heart cells, skin cells or nerve cells under the right conditions, or given the correct signals. They carry on the great potential for regenerative medicine, particularly in replacing cells in tissues with barely intrinsic renewal capacity, including the heart and nervous tissue.^14^

Mesenchymal stem cells (MSCs) are a promising alternative approach for healing human diseases. They primarily originate from the mesoderm and ectoderm during the early embryonic development and present mostly in adult tissues as bone marrow and adipose tissue, and foetal tissues and fluids as in the umbilical cord and amniotic fluid.^15^ The role of MSCs in the treatment of diseases depends on their ability to mend damaged tissue and suppress inflammation. In addition to their wide distribution, these cells are not immunogenic and can avoid immune cell recognition.^16^, ^17^

Adipose tissue–derived mesenchymal stem cells (ASCs) are unitary of the adult stem cells. They can be harvested from the stromal vascular fraction (SVF) of adipose tissues and have a great ability for multidirectional differentiation.^18^ Due to the mesodermal origin of ASCs, they have the capacity for self‐renewal and can also be differentiated into adipocytes, chondrocytes, myocytes, osteoblasts and neurocytes induced by a selective medium in vitro.^19^

White adipose tissue is the main source of ASCs, and the MSCs derived from it have a stronger antiapoptotic ability than that from brown adipose tissue. Among the advantages of ASCs is the easy access by subcutaneous lipoaspiration which is a much less painful procedure than harvesting bone marrow stem cells. They are harvested from autologous fat, unlike embryonic stem cells. So, their use is less associated with ethical controversies.^20^, ^21^

Adipose tissue–derived MSCs can pack trophic mediators into extracellular vesicles that can transport not only pro‐regenerative factors, but also mRNA and microRNA and even mitochondrial components over a long distance.^22^ They play as mediators for intercellular communication with neighbouring cells by exchanging these bioactive molecules or disseminating genetic contents towards distal organs. They are classified into exosomes, microvesicles and apoptotic bodies according to their respective size, origin, biogenesis and composition.^23^

Preclinical animal models showed that MSC‐derived MVs (MSC‐MVs) display pro‐angiogenic and cellular protective effects and can be used for tissue regeneration.^24^ Thus, MVs are promising therapeutic approach for various pathological conditions such as neural degeneration, liver fibrosis, kidney injury and myocardial infarction.^25^ Thus, the purpose of our work was to compare the therapeutic effect of ASCs versus MVs on the cerebellar damage induced by MSG.

## 2 MATERIALS AND METHODS

### 2.1 Animals

Forty adult healthy male Wistar rats (180–200 g) were kept at room temperature and light/dark (12/12 h) cycle, and allowed normal balanced diet and tap water. Experimental design and animal handling were approved by the local authorities at the Faculty of Medicine, Zagazig University, Zagazig, Egypt [Institutional Animal Care and Use Committee Zagazig University (ZU‐IACUC)].

### 2.2 Chemicals

Monosodium glutamate was supplied in the form of white powder. It was purchased from Cairo Pharma Co., Egypt.

### 2.3 Experimental design

Rats were equally divided into four groups. Group I (Control group) included 10 rats that were subdivided into three subgroups: subgroup IA (4 rats) that received no treatment, subgroup IB (3 rats) that received 2 ml of 0.9% sodium chloride (NaCl), intraperitoneally, and subgroup IC (3 rats) that received 0.5 ml of AD‐MSCs (1 × 10^7^ cells/ml) dissolved in 0.5ml of phosphate‐buffered saline (PBS), intravenous through the caudal vein. Group II (MSG‐treated group) received 2 ml of 3.5 mg/g body weight/day of MSG dissolved in 2 ml of 0.9% NaCl, intraperitoneally, for 10 days.^26^ Group III (MSG/ASC‐treated group) Received MSG (as described in group II) for 10 days. On the 11th day, each rat had a single dosage of 0.5 ml of ASCs (1 × 10^7^ cells/ml) suspended in 0.5 ml of PBS, intravenous through the caudal vein. Specimens were taken 4 weeks after stem cell administration.^27^ Group IV (MSG/MV‐treated group) received MSG (as described in group II) for 10 days. On the 11th day, each rat received a single dose of 200 μg MVs diluted in 1 ml of PBS, intravenous through the caudal vein. Specimens were removed 1 week after MV administration.^25^ Three rats died (2 rats from group II and one rat from group IV). They were excluded from the experiment.

### 2.4 Body weight

At the beginning of the experiment, rats of all groups were weighed for calculating the dose of MSG.

### 2.5 General observation

During the experimental period, the general appearance of rats and the quantity of food intake were determined daily.

### 2.6 Motor behaviour assessment

#### 2.6.1 Accelerating rotarod test^28^

**FIGURE 1:**
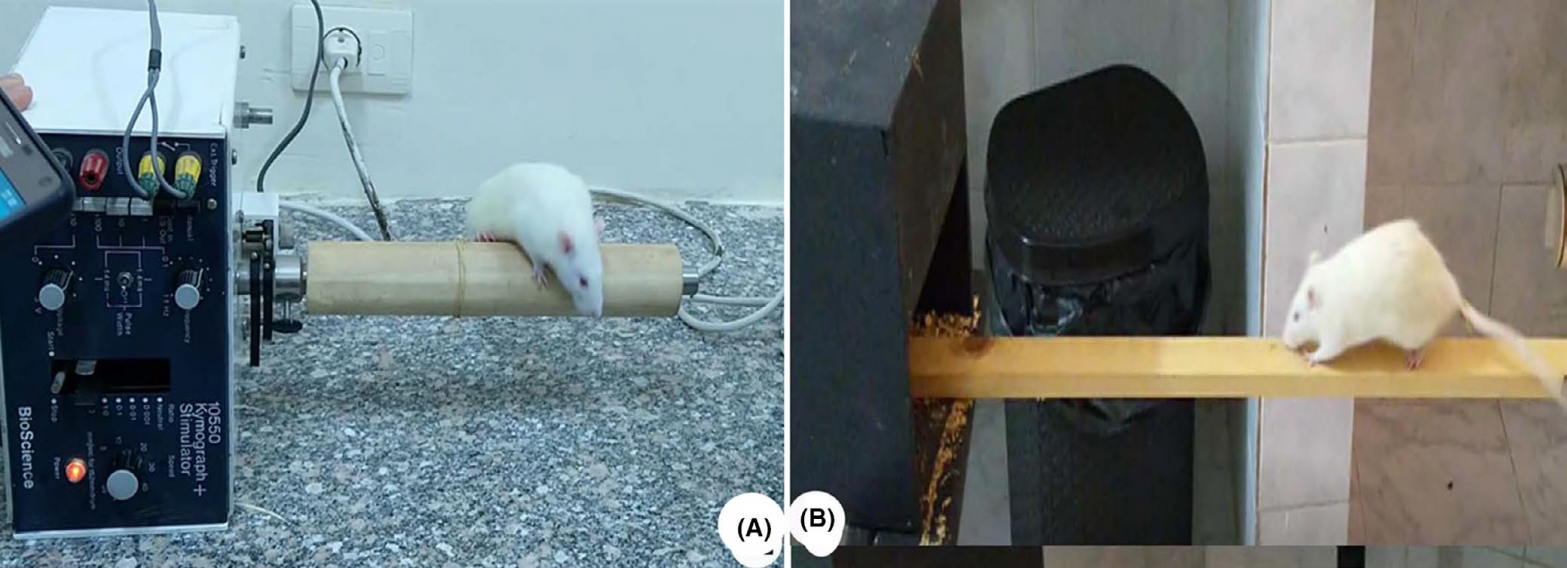
Motor behaviour assessment in the study groups. (A) Accelerating rotarod test and (B) Balance beam test

Rats were trained three times daily for one week before getting results. Each trial lasted for 5 min maximum, and rats were rested for 15 min between trials to avoid fatigue. The rats experienced linear acceleration from 4 to 40 rpm in 300 s. Latency to fall from rotarod was recorded in seconds (Figure 1A).

#### 2.6.2 Balance beam test^29^

This test examines the ability of the animal to remain upright and to walk on an elevated and fairly narrow beam (Figure 1B). Score from 0 to 5 was used as follows: 0: Falls off the beam immediately, 1: Falls off the beam before completing the walk, 2:Walks the beam, but is very instable, almost falling off, may pause one or more times, and/or takes more than 6 s, 3: Walks the beam, but is somewhat instable, may pause one or more times, takes more than 6 s to complete the walk, 4: Walks the beam, but is somewhat instable, completes the walk within 6 s, and 5: Walks the balance beam perfectly, does not need to check balance, does not pause, and completes the walk within 6 s.

### 2.7 Isolation and culture of adipose‐derived mesenchymal stem cells (ASCs)

Human adipose tissue was isolated from freshly subcutaneous adipose tissue samples obtained either from the abdomen or the inguinal fat. The adipose tissue was resected and placed into a labelled sterile tube containing 15 ml of phosphate‐buffered saline (PBS; Gibco/Invitrogen). Then, it was re‐suspended in 0.075% type collagenase II (sigma) in HBSS (approximately 2 ml/g) for enzymatic digestion, and incubated at 37°C for 60 min with shaking. At the end of digestion, 10% foetal bovine serum (FBS) (Gibco) was added to neutralize collagenase. The digested adipose tissue was passed through a 100‐μm filter to remove debris and centrifuged at 400 g for 10 min to obtain a cell pellet, and the erythrocytes were removed by treatment with erythrocyte lysis buffer. The cells were transferred to tissue culture flasks with Dulbecco Modified Eagle Medium (DMEM, Gibco/BRL) supplemented with 10% foetal bovine serum (Gibco/BRL), and after an attachment period of 24 h, non‐adherent cells were removed by a PBS wash. Attached cells were cultured in DMEM media supplemented with 10% foetal bovine serum FBS, 1% penicillin‐streptomycin (Gibco/BRL) and 1.25 mg/L amphotericin B (Gibco/BRL), and expanded in vitro. When large colonies developed (80%~90% confluence), cultures were washed twice with PBS and the cells were trypsinized with 0.25% trypsin in 1 mM EDTA (Gibco/BRL) for 5 min at 37℃. After centrifugation, cells were re‐suspended with serum‐supplemented medium and incubated in 50 cm^2^ culture flask (Falcon). The resulting cultures were referred to as first‐passage cultures and expanded in vitro until passage three.^30^

### 2.8 Identification and characterization of differentiated stem cells

ASC culture were known by their adhesiveness and fusiform shape, and by detection of positivity for CD105 (ASC surface markers) and negativity for CD106, CD14 and CD34 (hematopoietic markers) by flow cytometry.^31^

### 2.9 Labelling of stem cells with Paul Karl Horan 26 (PKH‐26)

ASCs were harvested and labelled with PKH26 dye (red fluorescence cell linker). Cells were centrifuged and washed twice in serum free medium then pelleted and suspended in dye solution.^32^

### 2.10 Detection of homing of stem cells

Cerebellar tissue was examined with a fluorescent microscope to detect and trace the cells stained with PKH26.^33^

### 2.11 Isolation of microvesicles

Microvesicles (MVs) were prepared by isolation of MSCs from the expanding medium, centrifugation, and taking the supernatant to be filtered several times to obtain the microvesicles. MSCs cultured for 24–48 h with DMEM medium. Then, the medium was collected, centrifuged at 1000 rpm = 20 rcf for 5 min, then at 2500 rpm = 126 rcf for 15 min, and filtered through 0.45 μm filter to remove the cellular debris. The MVs were prepared from the conditioned medium by ultracentrifugation (thermo scientific ultracentrifuge) at 100,000 g for 1 h at 4°C to pellet microvesicles in Medical Biochemistry Department, Kasr Al‐Ainy Faculty of Medicine. The pellets were taken and suspended in PBS. They were stored at −80°C until further use.^34^

### 2.12 Identification and characterization of microvesicles

They were characterized by transmission electron microscopy (TEM) and assessment of CD63 positivity (extracellular vesicles surface marker) by flow cytometry.^35^

### 2.13 Labelling of microvesicles with Paul Karl Horan 26 (PKH‐26)

The microvesicles were harvested and labelled with PKH26 dye (red fluorescence cell linker). They were centrifuged and washed twice in serum free medium, then pelleted and suspended in dye solution.^36^

### 2.14 Detection of homing of microvesicles

Cerebellar tissue was examined with a fluorescent microscope to detect and trace the cells stained with PKH26.^33^

### 2.15 Serum sampling and tissue and preparation

Blood collection from the ophthalmic plexus was done. Serum samples were obtained by centrifugation of blood at 2000 rpm = 80 rcf for 20 min. Then, supernatant sera were stored at −20°C. Serum samples were stored and used for biochemical assays. Part of cerebellar tissue was examined for histological evaluation, and the remaining part was homogenized with 5–10 ml cold buffer (50 mM potassium phosphate, pH 7.4, 1 mM EDTA) per gram tissue. Then, the homogenate was centrifuged 4000 rpm = 323 rcf for 15 min at 4°C, and the supernatant frozen at −80°C for oxidative stress parameters evaluation.

### 2.16 Lipid peroxidation assay

Malondialdehyde (MDA), a marker of lipid peroxidation (LPO), was measured calorimetrically in cerebellar homogenate according to the method of^37^ by commercially available kit (Biodiagnostic). Furthermore, serum reactive oxygen species (ROS) was estimated enzyme‐linked immunosorbent assay (ELISA) kit according to the manufacturer's instructions (MyBioSource).

### 2.17 Measurement of total oxidant status (TOS)

Total oxidative status (TOS) was measured in the cerebellar homogenates using a (Rel Assay Diagnostics kit). The assay was calibrated with hydrogen peroxide (H_2_O_2_), and findings were expressed as μmol H_2_O_2_ eq. /L.

### 2.18 Antioxidants measurements in the cerebellar homogenate

The superoxide dismutase (SOD) activity in U/g cerebellar tissue was measured by the method of Ref. [38]. Reduced glutathione (GSH) level was determined according to the method of Ref. [39]. Also, glutathione reductase (GR) activity was measured as described by.^40^ All the above kits produced from Biodiagnostic.

### 2.19 Estimation of serum total antioxidant capacity (TAC)

The serum total antioxidant capacity (TAC; Biodiagnostic) according to Ref. [41].

### 2.20 Real‐time analyses apoptotic genes in cerebellum

Total RNA was extracted from cerebellar tissue homogenate using the RNeasy extraction kit (Qiagen). The cDNA was obtained from extracted total RNA (5 μg) with 1 μl (20 pmol) antisense primer and 0.8 μl superscript AMV reverse transcriptase for 60 min at 37°C. The relative expression of mRNA was measured using SYBR Green method by Applied Biosystems. The sequences of the primers used in these assays were presented in Table 1. Quantitative real‐time PCR (qPCR) was performed in a total 25 μl reaction volume consisting of 2× SYBR Green PCR Master Mix (Applied Biosystems), 900 nM of each primer and 3 μl of cDNA. The PCR protocol was as follows: one cycle at 50°C for 2 min, one cycle at 95°C for 10 min, 40 cycles of denaturation at 95°C for 15 s and annealing/extension at 60°C for 10 min. The relative gene expression was calculated by normalizations of each target gene caspase 3, caspase 8, and caspase 9 to β‐actin gene (house‐keeping gene) by comparative Ct method^42^ (Table 1).

**TABLE 1:** Primers sequences used for real‐time polymerase chain reaction

Gene	Primer sequences	Annealing temperature	product size
Caspase 3	Forward primer: 5′‐CAGAGCTGGACTGCGGTATTGA‐3′	60°C	320
	Reverse primer:5′‐AGCATGGCGCAAAGTGACTG‐3′		
Caspase 8	Forward primer:5′‐CCCCACCCTCACTTTGCT‐3′	60°C	303
	Reverse primer:5′‐GGAGGACCAGGCACTTA‐3′		
Caspase‐9	Forward primer:5′‐AGCCAGATGCTGTCCCATAC‐3′	55°C	132
	Reverse primer:5‐CAGGAGACAAAACCTGGGAA‐3		
β‐Actin	Forward primer:5‐GAG AGG GAA ATC GTG CGT GAC‐3	55°C	453
	Reverse primer:5‐CAT CTG CTG GAA GGT GGA CA‐3		

### 2.21 Histological studies

#### 2.21.1 Light microscopic study

For light microscope study, specimens were fixed in a 10% formalin solution and processed to prepare 5‐μm‐thick paraffin sections for H&E^43^ and immunohistochemical stains for the detection of proliferating cell nuclear antigen (PCNA) and glial fibrillary acidic protein (GFAP) antibodies.

Immunohistochemical staining^44^: was done using the following:

1. Anti‐proliferating cell nuclear antigen (PCNA) antibody: mouse monoclonal antibody (Catalogue number M0879) obtained from DAKO life trade Egypt (sc‐56, lab vision, Santa Cruz Biotechnology Inc.). Positive cells show brown nuclear deposits.
2. Anti‐glial fibrillary acidic protein (GFAP) antibody: rabbit‐anti rat antibody (Catalog Number Z0334) obtained from DAKO life trade Egypt. Positive cells (glial cells such as astrocytes and ependymal cells) show brown cytoplasmic staining.

Immunohistochemical reaction was run out on paraffin sections of the cerebellar cortex using a streptavidin system. Paraffin sections were de‐paraffinized, rehydrated in descending grades of alcohol and incubated overnight with the primary monoclonal antibody. Sections were rinsed three times with PBS, then incubated for 1 h with peroxidase‐conjugated secondary antibody and washed three times with PBS. The reaction was developed with 0.05% diaminobenzidine (Dakopatts) as the substrate for peroxidase, and finally, the slides were counterstained with Mayer's haematoxylin. Negative control slides were prepared by replacing the primary antiserum with PBS. A tonsil was used as the positive control for PCNA. A brain slide was used as the positive control for GFAP.

#### 2.21.2 Electron microscopic study^45^

Specimens were immediately fixed in 2.5% glutaraldehyde for 2 h and postfixed in 1% osmium tetroxide buffered with 0.1 M phosphate buffer at pH 7.4 for 1 h. Then, they were dehydrated in ascending grades of alcohol and embedded in resin to prepare semithin and ultrathin sections using a Leica ultracut (UCT) (Glienicker). Semithin sections (1 μm thick) were stained with 1% toluidine blue and examined by light microscope.^46^ Ultrathin sections were stained with uranyl acetate and lead citrate.^47^ They were examined and photographed by JEOL JEM 2100 transmission electron microscope in Electron Microscope Research Laboratory Unit, Faculty of Agriculture, Mansoura University.

### 2.22 Morphometric analysis

The following parameters were measured: Number of Purkinje cells in H&E‐stained sections, number of PCNA immunoreactive cells and area percentage of GFAP immunoreactivity. Measurement was done using “Leica Qwin 500” software image analyzer computer system (Leica image system Ltd) at the Pathology Department, Faculty of Dental Medicine, Cairo University. Ten non‐overlapping fields were randomly chosen for each section at ×400 magnification.

### 2.23 Statistical analysis

Data management and statistical analysis were done using SPSS version 25 (IBM). Numerical data were summarized as means and standard deviations. Comparisons between the study groups were done using one‐way ANOVA test. Post hoc analysis was done using Bonferroni's method. All statistical tests were two‐sided. *p* values <0.05 were considered significant.^48^

## 3 RESULTS

### 3.1 General observation

During the experiment, the control rats were awake and alert while the MSG‐treated rats were weak and lethargic and gradually improved after ASC and MV treatment.

### 3.2 Motor behaviour assessment

#### 3.2.1 Accelerating rotarod test

Group II showed gradual deterioration in the latency to fall from the rotarod throughout the time of the experiment compared to the control group. Nevertheless, groups III and IV showed gradual deterioration through the 1st 2 weeks of assessment followed by some improvement along the remaining time of the experiment but did not come back to baseline values.

#### 3.2.2 Balance beam test

Rats in the control group walked easily and achieved a mean score of 5 while those in MSG‐treated group were unstable and took longer to finish walking, with a mean score of 2. However, rats in group III (ASCs) showed some improvement as they were slightly unstable, but could finish walking in time with an average score of 4. Moreover, group IV (MVs) showed some improvement but less than group III with an average score of 3.

### 3.3 Identification and characterization of differentiated stem cells

**FIGURE 2:**
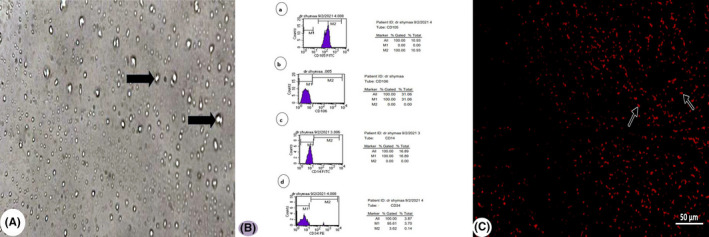
Characterization and homing of adipose tissue–derived mesenchymal stem cells (ASCs). (A) Culture of ASCs. Note, their adhesiveness and fusiform shape (arrow). (B) Flow cytometry of ASC surface markers, cells are positive for CD105 (a) and negative for CD106, CD14 and CD34 (b, c and d). (C) PKH26‐positive ASCs (arrow) in the cerebellar tissue (Fluorescent microscope ×200)

Adipose tissue–derived mesenchymal stem cells (ASCs) showed adhesiveness and fusiform shape in culture (Figure 2A). They were positive for CD105 (ASC surface markers) and negative for CD106, CD14 and CD34 (hematopoietic markers) by flow cytometry (Figure 2B).

### 3.4 Detection of homing of ASCs

Cerebellar tissue showed PKH26‐positive ASCs with a fluorescent microscope, indicating stem cell homing (Figure 2C).

### 3.5 Identification and characterization of MVs

**FIGURE 3:**
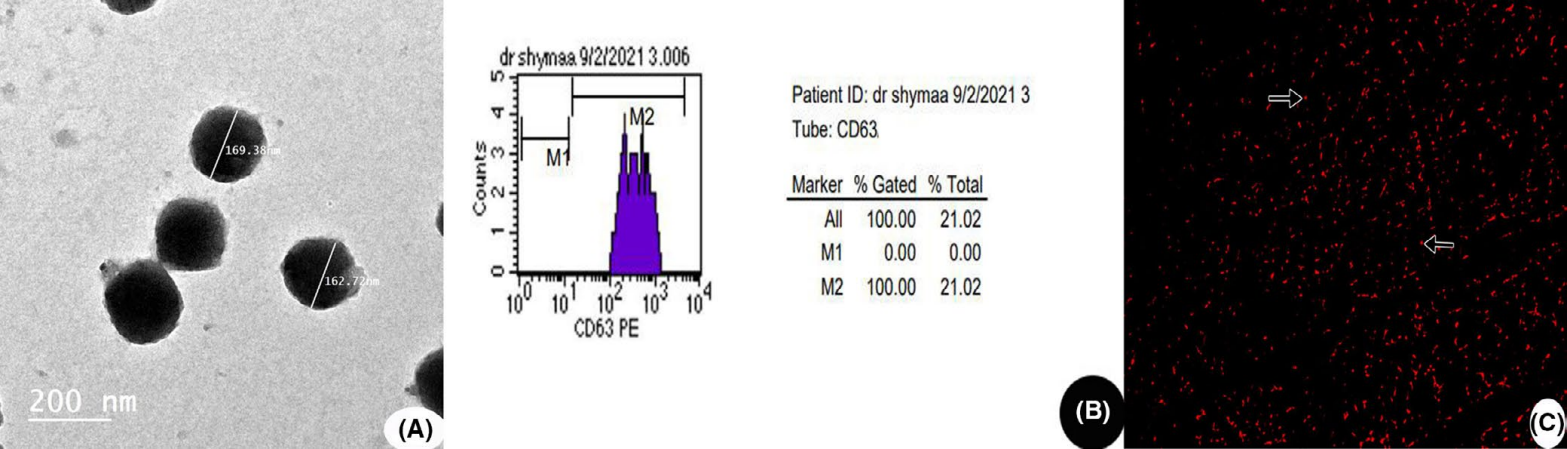
Characterization and homing of microvesicles (MVs). (A) Transmission electron micrograph of isolated microvesicles (arrow). They are heterogeneous, spheroid and variable in size. (B) Flow cytometry of microvesicles surface markers. They are CD63‐positive. (C) PKH26‐positive MVs (arrow) in the cerebellar tissue (Fluorescent microscope ×200)

By transmission electron microscope (TEM), MVs appeared as heterogeneous spheroid structures different in size (Figure 3A). They were positive for CD63 (extracellular vesicles surface marker) by flow cytometry (Figure 3B).

### 3.6 Homing of microvesicles

Cerebellar tissue showed PKH26‐positive MVs with a fluorescent microscope, indicating microvesicles homing (Figure 3C).

### 3.7 Oxidative stress parameters (Cerebellar MDA, lipid peroxidation marker and TOS):

Our findings showed significant differences between the four groups (*p* < 0.001). Post hoc analysis revealed significant increases of cerebellar MDA levels in group II (4.4 ± 0.2) compared to all other groups. In addition, significant decreases of MDA in group III followed by group IV; however, this difference was statistically non‐significant (*p* = 0.06 & *p* = 0.13 respectively). On the contrary, SOD activity in cerebellar tissue demonstrated a significant difference between the four groups (*p* = 0.004). Post hoc analysis revealed significant increases of cerebellar SOD levels in group III followed by group IV (29.6 ± 2.6 & 27.3 ± 2.8 respectively) compared to group II, with no significant differences between two groups (*p* = 0.64). Regarding cerebellar GSH concentrations, our data showed significant differences between study groups, with increased levels in group III followed by group IV in comparison with group II (*p* < 0.001 for each). Noteworthy, there were no significant differences of GR activity between groups. However, post hoc analysis revealed significant decreases of GR activity in group II compared to control group only. There were significant differences of serum TAC between groups (*p* < 0.001). Deeper analysis by post hoc showed a significant decrease of serum TAC in groups II, III and IV compared to group I (*p* < 0.001, *p* = 0.02, *p* = 0.03 respectively; Table 2).

**TABLE 2:** Oxidative stress parameters in the study groups

		Group I	Group II	Group III	Group IV	*p*‐value
MDA(nmol/g tissue)	Mean ± SD	1.7 ± 0.08	4.4 ± 0.2	2.1 ± 0.09	2.3 ± 0.1	<.001
Post hoc		2,3,4	1,3,4	1,2	1, 2	
TOS umo H_2_O_2_ equivl/l		20.2 ± 1.0	41.5 ± 2.1	17.2 ± 1.1	18.7 ± 1.3	<.001
Post hoc		2,3,4	1,3,4	1,2	1, 2	
SOD (U/g)		30.5 ± 2.6	22.4 ± 2.3	29.6 ± 2.6	27.3 ± 2.8	.004
Post hoc		2,3,4	1,3,4	2,3,4	1, 2,3	
GSH mmol/g tissue		8.9 ± 0.2	4.2 ± 0.3	9.3 ± 0.4	6.4 ± 0.7	<.001
Post hoc		2,3,4	1,3,4	2,3,4	1, 2,3	
GR (U/g)		5.7 ± 1.6	3.0 ± 1.2	4.3 ± 1.9	4.9 ± 1.8	.18
Post hoc		2	1			
TAC(Mm/l)		0.8 ± 0.04	0.01 ± 0.02	0.67 ± 0.09	0.65 ± 0.09	<.001
Post hoc		2,3,4	1,3,4	1,2,4	1,2,3	

Note

One‐way ANOVA was used. All post hoc comparisons were Bonferroni adjusted. 1; sig. diff. from G I 2; sig. diff. from G II 3; sig. diff. from G III 4; sig. diff. from G IV.

Abbreviation: GSH, reduced glutathione; GR, Glutathione reductase; MDA, malondialdehyde; SOD, superoxide dismutase; TAC, total antioxidant capacity; TOS, total oxidant status.

### 3.8 Effect of ASCs and MVs on Apoptosis gene expression

The data of the current research demonstrated significant differences between the four groups regarding apoptotitic gene expression caspases 3, 8 and 9 (*p* < 0.001 for each). MNG increased the expression of these genes in the cerebellar tissue compared with the control group. This was significantly restored by ASC and MV treatment for all caspases genes expression levels. Furthermore, there was no significant dereferences concerning of caspase gene expression values between ASC‐ and MV‐treated groups (Table 3). Thus, these finding shed the light on antiapoptotitic effects of ASCs and MVs.

**TABLE 3:** Relative apoptosis gene expression in the study groups

		Group I	Group II	Group III	Group IV	*p*‐value
Caspase 3	Mean ± SD	1.4 ± 0.1	3.9 ± 0.8	1.6 ± 0.4	1.8 ± 0.5	<.001
Post hoc		2,3	1,3,4	1,2,4	2	
Caspase 8		0.8 ± 0.04	2.7 ± 0.6	1.3 ± 0.2	1.4 ± 0.2	<.001
Post hoc		2,3,4	1,3,4	1,2	1,2	
Caspase 9	Mean ± SD	1.2 ± 0.3	5.1 ± 0.6	2.8 ± 0.7	2.6 ± 0.6	<.001
Post hoc		2,3,4	1,3,4	1,2	1,2	

Note

One‐way ANOVA was used. All post hoc comparisons were Bonferroni adjusted. 1; sig. diff. from G I 2; sig. diff. from G II 3; sig. diff. from G III 4; sig. diff. from G IV.

### 3.9 Histological results

Histological examination of the cerebellar sections of subgroups IA, IB and IC revealed no difference. Therefore, results of subgroup IA were represented as the control group.

#### 3.9.1 Light microscope results

**FIGURE 4:**
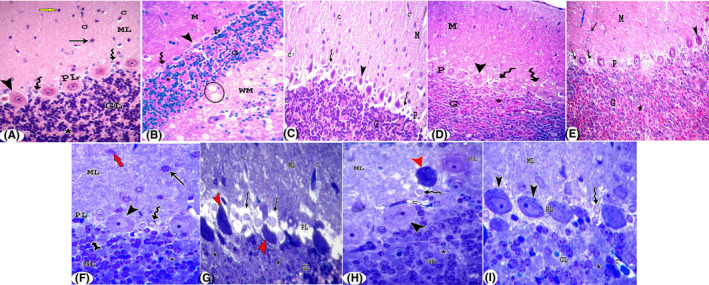
Sections in the cerebellar cortex stained with H&E (A–E) and toluidine blue (F–I). (A) Control group shows molecular (ML), Purkinje (PL) and granular (GL) layers. The molecular layer (ML) contains basket cells (black arrow) and smaller stellate cells (blue arrow). Blood capillaries (C) with pericapillary spaces are seen. The Purkinje cell layer is formed of a single layer of large pyriform Purkinje neuronal cells (arrowhead) with large pale nuclei and astrocytes (zigzag arrow). The granular layer shows closely packed small granule cells (GL) with pale areas of cerebellar islands (*) in between. In MSG‐treated group (B, C), Purkinje cells appear dark and shrunken (arrowhead). Many astrocytes are seen (zigzag arrow). Cerebellar islands are widened (*). The white matter (WM) shows degenerated neuropil (ellipse) (B). Shrunken Purkinje cells (arrowhead) appear with small dark nuclei and perineural spaces. Many astrocytes are seen (zigzag arrow). Many capillaries (C) with pericapillary spaces appear in the molecular layer (c). Group III shows Purkinje cells with architecture similar to that of the control group (arrowhead). Many astrocytes are seen (zigzag arrow) (D). In group IV, Purkinje cells have architecture comparable to that of the control group. However, few cells appear distorted (arrowhead). Several Bergmann protoplasmic astrocytes are seen (zigzag arrow) (E). (H&E ×400). (F) In the control group, the molecular layer (ML) contains basket cells (black arrow) and smaller stellate cells (red arrow). The Purkinje cell layer is formed of a single layer of large pyriform Purkinje neuronal cells with large central vesicular nuclei (arrow head). Bergmann protoplasmic astrocytes (zigzag arrow) are seen. The granular layer shows closely packed granule cells (bifid arrow) with patches of heterochromatin in their nuclei and cerebellar islands (*) in between. In MSG‐treated group (G), Purkinje cells are dark, distorted with ill‐defined nuclei (red arrowhead) and perineural spaces. Many astrocytes are seen (zigzag arrow). In group III (H), Purkinje cells have architecture similar to that of the control group (arrowhead). However, few cells appear dark and distorted (red arrowhead). Many Bergmann protoplasmic astrocytes are seen (zigzag arrow). In group IV (I), Purkinje cells show an architecture comparable to that of the control group (arrowhead). Many Bergmann protoplasmic astrocytes are seen (zigzag arrow). The granular layer show widened cerebellar islands (*). (Toluidine blue ×1000)

Haematoxylin and eosin–stained cerebellar sections of the control group revealed rat cerebellar cortex with its three layers: molecular, Purkinje and granular layers. The molecular layer contained basket cells and smaller stellate cells. Blood capillaries with pericapillary spaces were seen. The Purkinje cell layer was formed of a single layer of large pyriform Purkinje neuronal cells with large pale nuclei, and astrocytes. The granular layer showed closely packed small granule cells with pale areas of cerebellar islands in between (Figure 4A). In MSG‐treated group, Purkinje cells appeared dark and shrunken. Many astrocytes were seen. Cerebellar islands were widened. The white matter showed degenerated neuropil (Figure 4B,C). Group III showed Purkinje cells with architecture similar to that of the control group (arrowhead). Many astrocytes were seen (Figure 4D). In group IV, Purkinje cells had architecture comparable to that of the control group. However, few cells appeared distorted. Many astrocytes were seen (Figure 4E).

Semithin sections in the control group showed that the molecular layer contained basket cells and smaller stellate cells. The Purkinje cell layer was formed of a single layer of large pyriform Purkinje neuronal cells with large central vesicular nuclei. Bergmann protoplasmic astrocytes were seen. The granular layer showed closely packed granule cells with patches of heterochromatin in their nuclei and cerebellar islands in between (Figure 4F). At MSG‐treated group, Purkinje cells were dark, distorted with ill‐defined nuclei and peripheral spaces. Many astrocytes were seen (Figure 4G). In group III, Purkinje cells had architecture similar to that of the control group. Yet, few cells appeared dark and twisted. Many astrocytes were seen (Figure 4H). In group IV, Purkinje cells showed an architecture comparable to that of the control group. Many astrocytes were seen. The granular layer showed widened cerebellar islands (Figure 4I).

**FIGURE 5:**
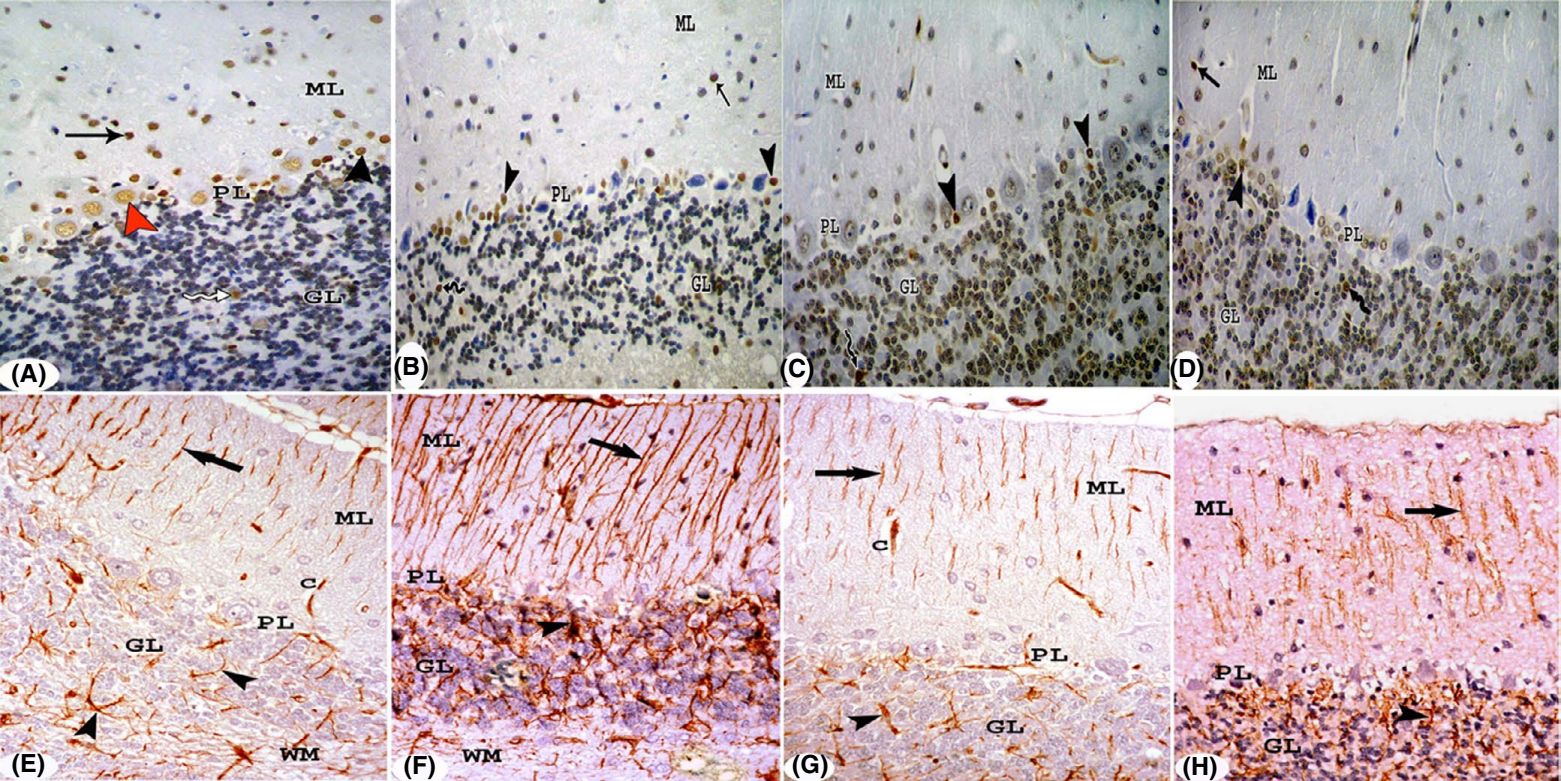
(A–E) Immunohistochemical localization of PCNA in sections of the cerebellar cortex. In the control group (A), intense immune reaction to PCNA in the nuclei of cells in the molecular layer (arrow), Purkinje cells (red arrowhead) and astrocytes (black arrowhead) in Purkinje cell layer, and few cells in the granular layer (zigzag arrow). In group II (B), the immune reaction is detected in few nuclei in the molecular layer (arrow), many astrocytes in Purkinje cell layer (black arrowhead) and few cells in the granular layer (zigzag arrow). However, in group III (C), the immune reaction is seen in the nuclei of few astrocytes in Purkinje cell layer (black arrowhead) and cells in the granular layer (zigzag arrow). Group IV (D) show positive reaction in the nuclei of a few cells in the molecular layer (arrow), many astrocytes in Purkinje cell layer (black arrowhead) and cells in the granular layer (zigzag arrow). (Immuneperoxidase ×400). (E–H): Immunohistochemical localization of GFAP in sections of the cerebellar cortex showing: Control group (E), in the molecular layer (ML), Bergmann‐glia or modified astrocytes (arrow) are perpendicular to the surface and parallel to each other while in the granular layer (GL), true astrocytes appear irregular (arrow head). Group II (F) shows an apparently increased GFAP‐positive astrocytes in the molecular (arrow) and granular (arrow head) layers. However, in group III (G), positive GFAP astrocytes are seen in the molecular (arrow) and granular (arrowhead) layers. Group IV (H) shows GFAP immune reactivity in Bergmann‐glia (arrow) and true astrocytes (arrowhead). (Immuneperoxidase ×400)

Immunohistochemical localization of PCNA in the control group revealed intense immune reaction to PCNA in the nuclei of cells in the molecular layer, Purkinje cells and astrocytes in Purkinje cell layer, and few cells in the granular layer (Figure 5A). In group II, the immune reaction was detected in few nuclei in the molecular layer, many astrocytes in Purkinje cell layer and few cells in the granular layer (Figure 5B). However, in group III, the immune reaction was seen in the nuclei of few astrocytes in Purkinje cell layer and cells in the granular layer (Figure 5C). Group IV showed positive reaction in the nuclei of a few cells in the molecular layer, many astrocytes in Purkinje cell layer and cells in the granular layer (Figure 5D).

Immunohistochemical localization of GFAP in the control group revealed that Bergmann‐glia or modified astrocytes were perpendicular to the surface and parallel to each other in the molecular layer, while in the granular layer, true astrocytes appeared irregularly (Figure 5E). Nevertheless, group II showed an apparently increased GFAP‐positive astrocytes in the molecular and granular layers (Figure 5F). In group III, GFAP‐positive astrocytes were seen in the molecular and granular layers. They were nearly similar to that of the control group (Figure 5G). Group IV showed GFAP immune reactivity in Bergmann‐glia and true astrocytes (Figure 5H).

#### 3.9.2 Electron microscope results

**FIGURE 6:**
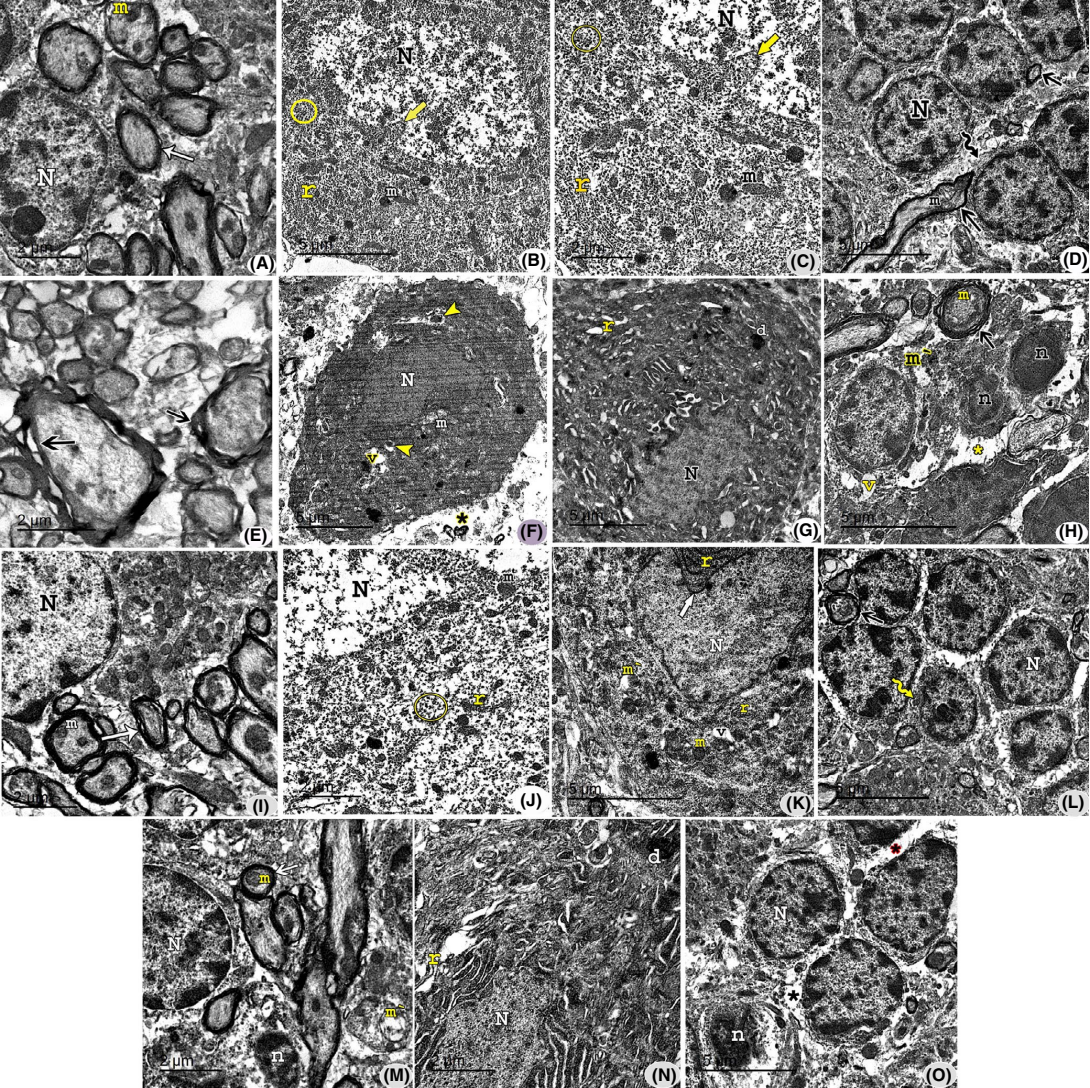
Transmission electron micrographs of sections in the cerebellar cortex; (A–D): Control group, (E–H): Group II, (I–L): Group III and (M–O): Group IV. The molecular layer (A) shows normal myelinated axons (white arrow) containing mitochondria (m). A part of the granular layer is seen with a granule cell containing nuclei (N) with heterochromatin clumps. The Purkinje layer (B & C): Purkinje cell contains euchromatic nucleus (N) with an indentation in the nuclear membrane (yellow arrow). The cytoplasm contains cisternae of rough endoplasmic reticulum (r), mitochondria (m) and free ribosomes (circle). (C): A higher magnification of fig. 6(b). In the granular layer (D), multiple granule cells contain nuclei (N) with heterochromatin clumps and thin rim of cytoplasm (zigzag arrow). Myelinated nerve fibres reveal regular compact myelin sheathes (black arrow) and contain mitochondria (m). (E) Myelinated nerve fibres reveal separation in myelin sheathes (black arrow). Purkinje cell appears dark with irregular ill‐defined nucleus (N). The cytoplasm shows vacuoles (v), mitochondria (m) with disrupted cristae and dense bodies (yellow arrowhead). A perineural space (*) can be seen indicating degenerated neuropil (F). Another Purkinje cell containing an irregular nucleus (N), dilated cisternae of rough endoplasmic reticulum (r) and dense bodies (G). (H) Some granule cells reveal shrunken dark nuclei (n). Others shows vacuoles (v) and mitochondria with disrupted cristae (m`). Perineural spaces (*) are seen. Myelinated fibres with separated myelin sheath (black arrow) and degenerated mitochondria (m) are also seen. (I) Myelinated axons (arrow) contain mitochondria (m). A part of the granular layer can be detected with granule cell nucleus (N) containing heterochromatin clumps. A part of Purkinje cell reveals euchromatic nucleus (N) with an indentation of the nuclear membrane (yellow arrow). The cytoplasm contains cisternae of rough endoplasmic reticulum (r), mitochondria (m) and free ribosomes (circle) (J). Another Purkinje cell shows an irregular nucleus with an indentation of the nuclear membrane (white arrow). The cytoplasm contains rough endoplasmic reticulum (r), some mitochondria (m) with disrupted cristae (mˋ) and vacuoles (V) (K). (L) Multiple granule cells reveal clumps of heterochromatin in their nuclei (N) and thin rim of cytoplasm (zigzag arrow). A myelinated fibre with slightly separated myelin sheath (black arrow) can be seen. (M) Myelinated axons with intact myelin sheath (white arrow) are seen containing mitochondria (m). Few unmyelinated fibres contain vacuolated mitochondria (mˋ). A granule cell with heterochromatin clumps in its nucleus can be seen. Few granule cells appear with dark shrunken nucleus (n). In (N), A Purkinje cell appears with an irregular ill‐defined nucleus (n). The cytoplasm contains dilated cisternae of rough endoplasmic reticulum (r) and electron‐dense bodies (d). Note, myelinated axons (white arrow). In (O), multiple granule cells with heterochromatin clumps in their nuclei (N) and thin rim of cytoplasm. Few cells appear dark and shrunken (N)

Examination of ultrathin cerebellar sections of the control group showed the cortex with its three layers: the outer molecular, middle Purkinje and inner granular. The molecular layer showed normal myelinated axons with mitochondria. A part of the granular layer was seen with granular cells containing nuclei with heterochromatin clumps (Figure 6A). The Purkinje cell layer showed a part of Purkinje cell, which had an euchromatic nucleus with an indentation in the nuclear membrane. The cytoplasm contained cisternae of rough endoplasmic reticulum, mitochondria and free ribosomes (Figure 6B,C). The granular layer showed multiple granule cells with clumps of heterochromatin in their nuclei and a thin rim of cytoplasm. Myelinated nerve fibres revealed regular compact myelin sheathes with mitochondria (Figure 6D).

In group II, in the molecular layer showed myelinated nerve fibres with separation in the myelin sheath (Figure 6E). Purkinje cell appeared dark with an ill‐defined nucleus. The cytoplasm showed vacuoles, mitochondria with disrupted cristae and dense bodies. A perineural space was seen indicating degenerated neuropil (Figure 6F). A part of Purkinje cell showed irregular nucleus, dilated cisternae of rough endoplasmic reticulum and dense bodies mostly lysosomes (Figure 6G). Some granule cells revealed shrunken dark nuclei. Others showed vacuoles and mitochondria with disrupted cristae. Perineural spaces were seen. Myelinated fibres with separated myelin sheath and degenerated mitochondria were also seen (Figure 6H).

In group III, the molecular layer showed some normal myelinated axons with mitochondria (Figure 6I). A part of Purkinje cell revealed euchromatic nucleus with an indentation of the nuclear membrane and the cytoplasm with cisternae of rough endoplasmic reticulum, mitochondria and free ribosomes (Figure 6J). Another Purkinje cell showed an irregular nucleus with an indentation of the nuclear membrane, and dilated rough endoplasmic reticulum, few mitochondria with disrupted cristae and vacuoles (Figure 6K). Multiple granule cells revealed clumps of heterochromatin in their nuclei and thin rim of cytoplasm. Myelinated fibres with separated myelin sheath were seen (Figure 6L).

Group IV showed myelinated axons with intact myelin sheath were seen included mitochondria and few unmyelinated fibres with vacuolated mitochondria. A granular cell with heterochromatin clumps in its nucleus was seen (Figure 6M). A Purkinje cell appeared with an irregular, ill‐defined nucleus, dilated cisternae of rough endoplasmic reticulum and electron‐dense bodies (Figure 6N). Multiple granule cells show nuclei (N) with heterochromatin clumps, and thin rim of cytoplasm. Few cells appeared dark and shrunken (Figure 6O).

#### 3.9.3 Morphometric and statistical results

The non‐significant difference was detected between subgroups IA, IB and IC. Therefore, the average of group I was used as a control to be compared with the other groups of the study.

**FIGURE 7:**
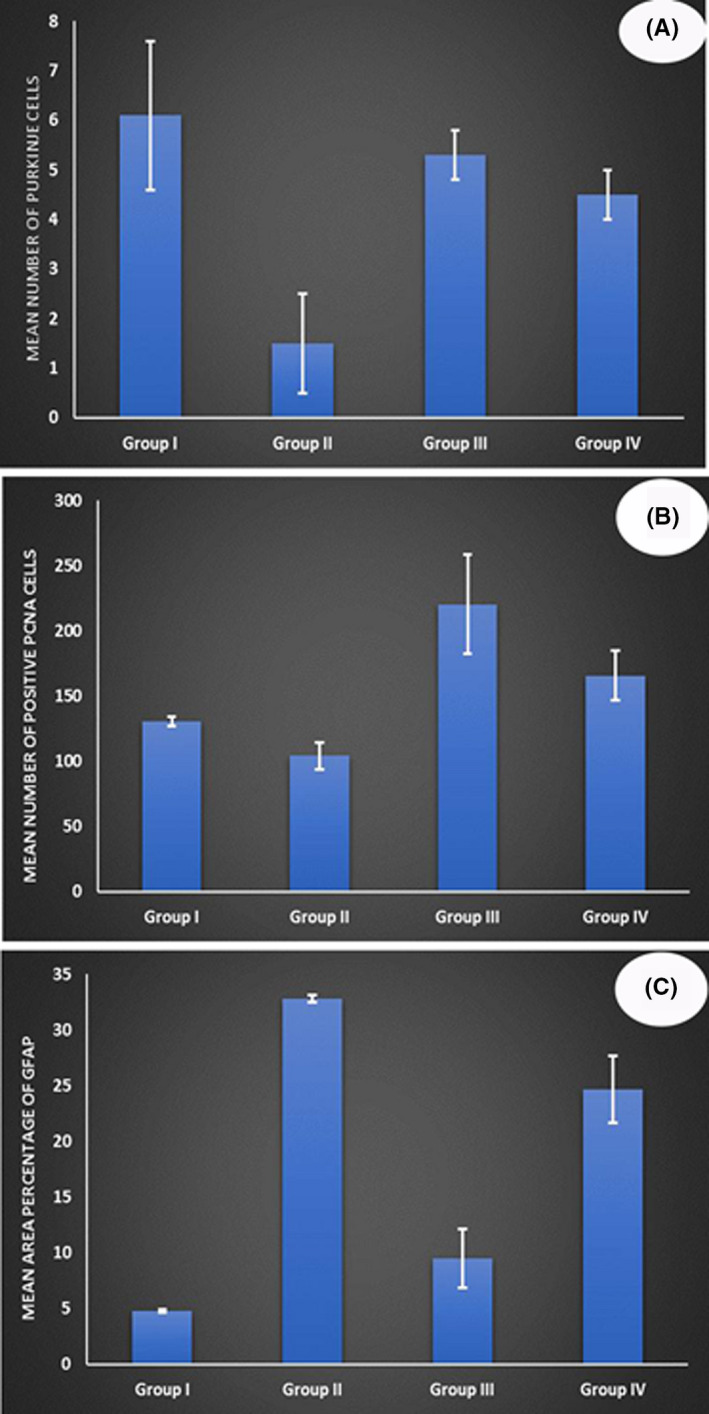
Morphometric results in the study groups. (A) Number of Purkinje cells, (B) Number of positive PCNA cells and (C) Area percentage of GFAP.

The number of Purkinje cells showed an overall significant difference between the four groups (*p*‐value <0.001). Post hoc analysis revealed that the number of Purkinje cells in group II (1.5) was significantly lower than all other groups. The number of Purkinje cells in group I was significantly higher (6.1) than in group IV (4.5). There were no significant differences between group III, group I and group IV (Table 4 and Figure 7A).

**TABLE 4:** Number of Purkinje cells in the study groups

		Group I	Group II	Group III	Group IV	*p*‐value
Number of Purkinje cells	Mean ± SD	6.1 ± 1.5	1.5 ± 1	5.3 ± 0.5	4.5 ± 0.5	<.001
Post hoc		2,4	1,3,4	2	1, 2	

Note

One‐way ANOVA was used. All post hoc comparisons were Bonferroni adjusted. 1; sig. diff. from G I 2; sig. diff. from G II 3; sig. diff. from G III 4; sig. diff. from G IV.

The number of positive PCNA cells showed an overall significant difference between the four groups (*p*‐value <0.001). Post hoc analysis revealed that the number of positive PCNA cells in group III (220.5) was significantly higher compared to all other groups. Likewise, the number of positive PCNA cells in group VI was significantly higher (165.7) than groups I (130.5) and II (104.1). There was a borderline significant difference between groups I and II, with a higher number of positive PCNA cells in group I (Table 5 and Figure 7B).

**TABLE 5:** Number of positive PCNA cells in the study groups

		Group I	Group II	Group III	Group IV	*p*‐value
Number of positive PCNA cells	Mean ± SD	130.5 ± 3.7	104.1 ± 10.5	220.5 ± 38.3	165.7 ± 18.9	<.001
Post hoc		3,4	3,4	1,2,4	1,2,3	

Note

One‐way ANOVA was used. All post hoc comparisons were Bonferroni adjusted. 1; sig. diff. from G I 2; sig. diff. from G II 3; sig. diff. from G III 4; sig. diff. from G IV.

Area percentage of GFAP immune reactivity showed an overall significant difference between the four groups (*p* < 0.001). Post hoc analysis revealed that the area percentage of GFAP in group II (32.8) was significantly higher compared to all other groups. Also, in group IV, it was significantly higher (24.7) than groups I (4.8) and III (9.5). No substantial difference was reported between groups I and III (Table 6 and Figure 7C).

**TABLE 6:** Area percentage of GFAP in the study groups

		Group I	Group II	Group III	Group IV	*p*‐value
Area percentage of GFAP	Mean ± SD	4.8 ± 0.1	32.8 ± 0.3	9.5 ± 2.6	24.7 ± 3	<.001
Post hoc		2,4	1,3,4	2,4	1,2,3	

Note

One‐way ANOVA was used. All post hoc comparisons were Bonferroni adjusted. 1; sig. diff. from G I 2; sig. diff. from G II 3; sig. diff. from G III 4; sig. diff. from G IV.

## 4 DISCUSSION

MSG is considered the most widely used food additive. While having huge benefits to the food industry, the ubiquitous use of this food additive could have negative consequences for public health.^6^ In the present study, assessment of cerebellar function revealed deterioration of the motor function in MSG‐treated rats, compared to the control group, manifested by a gradual deterioration in the latency to fall from the retard and usability and longer time to finish walking, with a mean score of 2, by balance beam test. Similar results were reported by Aminuddin et al.^49^ and Araujo et al.^50^ In agreement,^51^ found that increase the level of MSG in the synaptic cleft region results in excessive glutamate receptor activation with persistent depolarization (excitotoxicity) producing metabolic and functional exhaustion of the affected neurons leading to neural necrosis, cerebellar damage and motor function deterioration.

In the current work, MSG‐treated group revealed degeneration of some Purkinje cells. Few cells appeared dark and shrunken with small, dark and ill‐defined nuclei. These results were confirmed statistically as the number of Purkinje cells in the MSG group was significantly lower than all other groups. Similar results with an evidence of neuronal damage due to the neurotoxic effect of MSG were reported by Ibrahim et al.^52^ and Abdelwahed et al.^53^

In the same context, Gill et al.^54^ and Lau & Tymianski^55^ suggested that persistence of high glutamate concentration exceeding the neuronal capacity causes hyperactivation of glutamate receptors with continued depolarization and metabolic and functional depletion of the affected neurons. This results in an increase in the intracellular calcium levels leading to modification in the dendritic structure and interruption of the normal synaptic function. Likewise, activation of degraded calcium‐dependent enzymes and apoptotic pathways lead to neural necrosis. Previously, Mohamed et al.^56^ considered pyknotic nuclei in the cerebral cortex as irreversible condensation of chromatin in the nuclei of cells undergoing necrosis.

According to Fardian et al.,^57^ increased glutamate concentration induced cell death in the pyramidal cells of the cerebral cortex through autophagy by lysosomes. The uncontrolled lysosome activity led to internal destruction and ended in neural necrosis. Moreover, the neurotoxic effect of MSG can be mediated by a process of oxidative stress that leads to glutathione depletion and increased free radical formation resulting in oxidative glutamate toxicity.^58^, ^59^ In contrast to our results, Ashraf et al.^60^ reported that there was no change in the count of Purkinje cells but only a significant change in the inter Purkinje cell distance.

As regard group III, rats received ASCs intravenous, through the caudal vein. Other researchers used different approaches for ASC administration either by intrathecal injection into a rat model via lumbar puncture to deliver mesenchymal stem cells to the brain or transplantation of ASCs through the cerebrospinal fluid by inserting a needle into the subarachnoid space.^61^, ^62^

In the current work, administration of ASCs caused functional and structural improvement as there was a gradual deterioration in the motor function through the first 2 weeks of assessment followed by some improvement along the remaining time of the experiment but did not return to baseline values. Also, the Purkinje cells were nearly similar to the control. Myelinated nerve fibres appeared with intact myelin sheaths. These results could be attributed to the ability of ASCs to promote axonal regeneration, myelination and functional recovery as they can upregulate the expression of myelin proteins as myelin protein zero and myelin basic protein. Therefore, ASCs were applied to preclinical trials for treatment of CNS diseases such as Alzheimer's and Parkinson's diseases.^63^, ^64^ Some animal models have proven that ASCs have neuroprotection properties and provide support for axon regeneration in optic nerve transection and retinitis pigmentosa.^65^

The current study showed increased oxidative parameters, cerebellar MDA and TOS in the MSG‐treated group, which was reversed in ASC‐treated group and MV‐treated group. In contrast, the cerebellar antioxidant parameters, SOD and GSH levels or serum TAC concentrations were significantly decreased by MSG, which was more improved by ASC or MV treatment with better improvement with ASCs. However, there were no significant differences of GR activity between groups. Thus, the present research shed the light on oxidative stress caused by MSG, which can be reversed by either ASCs or MVs. These data could be justified by the possible therapeutic role of ASCs in neurodegenerative diseases was also explained by their ability to reduce the inflammatory response and glia cell activation. ADSCs can differentiate into cells with several neuronal and glial characteristics in vitro.^66^, ^67^, ^68^ Kim et al.^69^ suggested that this improvement could be due to activation of endogenous stem cell reproduction and differentiation or formation of new growth factors as vascular endothelial growth factor, fibroblast growth factors, hepatocyte growth factor, platelet‐derived growth factor, interleukin‐6, insulin‐like growth factors, epidermal growth factor and transforming growth factor‐β. These factors can decrease oxidative stress, suppress local immune responses and decrease cell death. In the same context, Korayem et al.^29^ used hematopoietic stem cells to attain improvement in the structure and function of the cerebellum.

MSCs can integrate into the central nervous system, migrate towards the areas where neurodegenerative processes occur, and rescue the degenerating cells through paracrine function of stem cells or cell trophic effect. The latter is a mechanism that accelerates neural differentiation and secretes neuroprotective factors to enhance neural regeneration.^70^

In the current study, administration of MVs to rats led to slight improvement in the motor function. Previously, the same dose of MVs demonstrated therapeutic effects in a rat model of ischaemia mediated by the paracrine action of MVs that promotes angiogenesis, neurogenesis and anti‐inflammatory processes to enhance recovery and reduce brain infarct size.^71^

In addition, group IV showed Purkinje cells with architecture nearly similar to that of the control group with few dark and distorted ones. These results were confirmed by morphometric and statistical results as the number of Purkinje cells in group IV was significantly lower than the control and no significant difference was found between groups III and IV. Herrera et al.,^72^ and Raisi et al.,^24^ reported that the MV anti‐inflammatory and regenerative mechanisms can be used as an alternative to omental adipose‐derived MSCs for the improvement of rat sciatic nerve and liver regeneration.

Various studies proved that the secreted factors from stem cell alone without the stem cell itself may cause tissue repair in various conditions. These factors, as secretive, microvesicles or exorcism can be found in the conditioned medium where stem cells were cultured. Stem cell‐derived conditioned medium has many advantages for regenerative medicine than stem cell alone. It contains tissue regenerative agents, various growth factors and cytokines, which will satisfy requirements of the damaged tissues. The secretome containing conditioned medium can be manufactured, freeze‐dried, packaged and transported more easily. Also, there are no rejection problems or infections because there are no cells in it.^69^, ^73^, ^74^

Also, MVs may be taken in the bidirectional exchange of genetic information between stem and injured cells. The transfer of gene products from injured cells may explain stem cell functional and phenotypic changes without the need for differentiation into tissue cells. On the contrary, the transfer of gene products from stem cells may reprogram injured cells to repair damaged tissues.^75^

Our study revealed a borderline significant difference in PCNA reaction between the control and MSG‐treated groups, with a higher number of positive PCNA cells in the control. Ino & Chiba^76^ found that PCNA mRNA is expressed not only in proliferating cells but also in non‐proliferating cells such as neurons. Also, positive expression of PCNA can be observed in neurons of the cerebellar cortex and the vestibular nuclei, while immunolabelling is impaired in the lateral geniculate nucleus and cerebral cortex in feline brains.^77^ In the same context, MSG was found to decrease PCNA immunoreactivity in spermatogonia and spermatocytes revealing increased apoptosis and decreased proliferation processes of the testicular germ cells.^78^, ^79^ On the contrary, Hegazy et al.^80^ showed high PCNA immunoreaction in the MSG‐treated cells that were explained by tendency of these cells to proliferate more to compensate degeneration and damage.

On the contrary, the immune expression of PCNA was increased in groups III and IV as compared to MSG‐treated group. These results were confirmed by morphometric and statistical results. Similarly, Cetinkaya et al.^81^ and Jiao et al.^82^ reported increased expression of PCNA and Ki67 in MSCs group. MSCs could markedly suppress the expression of P21, and promote the level of PCNA, thus improve function of ageing spleen and thymus and suppress oxidative stress.^83^ SCs and microvesicles could help in the proliferation and regeneration of degenerated cells.^84^, ^85^ Thus, they might be recommended as an alternative therapy for treating peripheral neuropathy.^86^

Our immunohistochemical results revealed intense reaction to GFAP in MSG‐treated group which was statistically confirmed by a significant increase in area percentage of GFAP immune reaction, compared to the control group. Sriram et al.^87^ and Baydas et al.^88^ reported that any mechanical, chemical or degenerative insult to the brain stimulates astrocyte proliferation and hypertrophy with increased GFAP synthesis resulting in vigorous astrogliosis. Expression of GFAP inhibits inflammatory response after injury and limiting the damage.^89^ In contrast to our results, previous researches reported toxic effect of MSG on the astrocytes themselves. Also, the toxic effects of reduced glutathione and reactive oxygen species accumulation on astrocytes have been reported,^90^, ^91^, ^92^, ^93^, ^94^ which confirmed our finding regarding the oxidative stress reported in the present work.

We found that overexpression of caspase 3, caspase 8 and caspase 9 genes in cerebellar tissues in the MSG group compared to control group, which restored by ASC or MV treatment. These findings are in accordance with.^95^ They proposed that cellular stress induced by MSG leads to apoptosis. Additionally, they reported that multiple and distinctive death programmes as caspase 3–dependent microvascular disruption mediated neuronal and glial cell death occur in the brain stem and disruption of blood‐brain barrier integrity as well as neural connectivity as a result of MSG exposure.^95^

On the contrary, the area percentage of GFAP reaction in groups III and IV were significantly lower than MSG group. In group VI, it was significantly higher than groups I and III. No significant difference was found between groups I and III. In the same context, Ou et al.^96^ and Korayem et al.^29^ detected GFAP‐positive astrocytes, 28 days after stem cell transplantation in rats exposed to middle cerebral artery occlusion. ASCs were suggested to inhibit the activation of glia and microglial and reduce the expression levels of pro‐inflammatory cytokines TNF‐α, IL‐1β and IL‐6 in the cerebellum.^97^, ^98^, ^99^

In conclusion, Monosodium glutamate (MSG) is the most commonly used food additive. While having huge benefits to the food industry, the ubiquitous use of MSG may have negative consequences for public health. In the present study, MSG induced degenerative changes in the cerebellar cortex and functional disturbance. Administration of ASCS and MVs were proved to have neuroprotective effects, possibly due to neural differentiation and paracrine, antiapoptotic, anti‐inflammatory and antioxidant effects. However, our results revealed better results in case of ASCs. Thus, ASCs and MVs can be promising in the treatment of neural diseases to rescue the degenerating cells. However, future clinical studies are required to assure whether this treatment strategy is clinically relevant to patients.

## CONFLICT OF INTEREST

There is no conflict of interest to declare.

## AUTHOR CONTRIBUTIONS

**Nehad Fahmy Mazen:** Conceptualization (equal); Data curation (equal); Methodology (equal); Resources (equal); Validation (equal); Visualization (equal); Writing – original draft (equal); Writing – review and editing (equal). **Eman Abdel‐Razik:** Conceptualization (equal); Supervision (equal). **Shimaa Ragab Desoky:** Conceptualization (equal); Writing – review and editing (equal). **Amal S. El‐Shal:** Conceptualization (equal); Investigation (equal).

## CODE AVAILABILITY

Leica Qwin 500 software image analyzer computer system (Leica image system Ltd; Cambridge, England).

## CONSENT TO PARTICIPATE

Not applicable.

## CONSENT FOR PUBLICATION

Not applicable.

## Data Availability

All data generated or analysed during this study are included in this published article.
